# Views on and experiences of electronic cigarettes: a qualitative study of women who are pregnant or have recently given birth

**DOI:** 10.1186/s12884-018-1856-4

**Published:** 2018-06-15

**Authors:** Katharine Bowker, Sophie Orton, Sue Cooper, Felix Naughton, Rachel Whitemore, Sarah Lewis, Linda Bauld, Lesley Sinclair, Tim Coleman, Anne Dickinson, Michael Ussher

**Affiliations:** 1 NIHR School for Primary Care Research and UK Centre for Tobacco and Alcohol Studies, Division of Primary Care, School of Medicine, University of Nottingham, Nottingham, NG7 2RD UK; 2 School of Health Sciences, University of East Anglia, Edith Cavell Building, Norwich, NR4 7TJ UK; 3 Division of Epidemiology and Public Health and UK Centre for Tobacco and Alcohol Studies and, University of Nottingham, Clinical Sciences Building 2, Nottingham City Hospital, Hucknall Road, Nottingham, NG5 1PB UK; 4 Institute for Social Marketing and UK Centre for Tobacco and Alcohol Studies, University of Stirling, Stirling, FK9 4LA UK; 5 Population Health Research Institute, St George’s, University of London, London, SW17 0RE UK

**Keywords:** Pregnancy, Postpartum, Electronic cigarettes, Qualitative, Interviews

## Abstract

**Background:**

Electronic cigarettes (ECs) are increasingly used for reducing or stopping smoking, with some studies showing positive outcomes. However, little is known about views on ECs during pregnancy or postpartum and previous studies have nearly all been conducted in the US and have methodological limitations, such as not distinguishing between smokers and ex/non-smokers. A greater understanding of this topic will help to inform both clinicians and EC interventions. We elicited views and experiences of ECs among UK pregnant or recently pregnant women.

**Methods:**

We conducted semi-structured telephone interviews, using topic guides, with pregnant or recently pregnant women, who were current or recent ex-smokers. To ensure broad views of ECs were obtained, recruitment was from several geographical locations and via various avenues of recruitment. This included stop smoking services, antenatal and health visitor clinics, a pregnancy website and an informal network. Participants were 15 pregnant and 15 postpartum women, including nine current EC users, 11 ex-users, and 10 never-users. Five women who were interviewed in pregnancy were later interviewed in postpartum to explore if their views had changed. Audio data was transcribed verbatim and framework analysis was applied.

**Results:**

Five main themes emerged: motivations for use (e.g., for stopping or reducing smoking), social stigma (e.g., avoiding use in public, preferring ‘discrete’ NRT), using the EC (e.g., mostly used at home); consumer aspects (e.g., limited advice available), and harm perceptions (e.g., viewed as less harmful than smoking; concerns about safety and addiction).

**Conclusions:**

ECs were viewed positively by some pregnant and postpartum women and seen as less harmful than smoking and useful as aids for reducing and stopping smoking. However, due to perceived social stigma, some women feel uncomfortable using ECs in public, especially during pregnancy, and had concerns about safety and nicotine dependence. Health professionals and designers of EC interventions need to provide women with up-to-date and consistent information and advice about safety and dependence, as well as considering the influence of social stigma.

**Electronic supplementary material:**

The online version of this article (10.1186/s12884-018-1856-4) contains supplementary material, which is available to authorized users.

## Background

Maternal smoking in pregnancy is the main preventable cause of morbidity and death among women and infants [1, 2]. In high income countries smoking rates in pregnancy tend to be between 10 and 13% [3–5]. Despite nicotine replacement therapy’s (NRT) effectiveness for non-pregnant smokers [6], in pregnancy it has been shown to be no more effective than placebo [7]. This is most likely due to low adherence, as well as insufficient dosage because of higher nicotine metabolism in pregnancy [7]. Financial incentives have shown promise for smoking cessation in pregnancy, but there are ethical challenges [8]; for example, there is the possibility that women might falsify their smoking status in order to receive the incentive. Electronic cigarettes (ECs) may have a role to play, as they potentially address both nicotine addiction and the behavioural aspects of smoking.

ECs are increasingly used in many countries, including the US [9], and are the most popular cessation aid in the UK [10]. It has been argued that the potential benefits of ECs probably outweigh harms, and that ECs may have an important role in harm reduction [11–13]. As regards the prevalence of EC use in pregnancy, one recent US study observed that 53% of 103 pregnant smokers reported having tried ECs [14] and a nationally representative US survey found that 29% of 100 pregnant smokers were currently using ECs [15]. The efficacy and safety of using ECs in pregnancy is untested. Nicotine has the potential to show teratogenic effects in the fetus [16], but there is no evidence for this. ECs share a similar pharmacokinetic profile to NRT, which does not appear to have detrimental effects on offspring at birth or 2 years postpartum, compared with placebo [17, 18]. In the UK, it is recommended that pregnant women should not be discouraged from using ECs for stopping smoking if they have struggled to quit without a cessation aid [19]. However, due to lack of evidence on efficacy and safety, in Australia [20] and the US [21] women are advised not to use ECs in pregnancy.

While several studies have investigated views on EC use in pregnancy, these have significant limitations such as the inclusion of women who were neither pregnant nor postpartum [22, 23], investigation of a restricted set of views [22, 24], recruiting from one geographical area [25, 26], and not distinguishing between smokers and ex/non-smokers [24, 27, 28]. Moreover, only one of these studies [25] included postpartum women. In addition, besides one online study [28], they were all conducted in the US. Given these limitations, there are significant knowledge gaps in this area. We attempted to overcome the limitations of previous studies by conducting a UK-based qualitative, in-depth exploration of pregnant and postpartum women’s views and experiences of ECs.

## Methods

A qualitative descriptive methodology was chosen, allowing an in-depth approach to eliciting a rich description of views and experiences for individual women. This approach also enabled the exploration of differences between individuals. A purposive sampling strategy was adopted to ensure the inclusion of a wide variation of participants to maximise the chances that a full spectrum of views on e-cigarette use in pregnancy were captured. Semi-structured telephone interviews were conducted with 30 UK pregnant or recently pregnant (given birth in last 6 months) women, self-reporting as current smokers (smoked in past 30 days) or recent ex-smokers (stopped smoking in 3 months before pregnancy or after finding out about pregnancy). Women were recruited with various experience of ECs and at different gestations. Following their initial interview, pregnant participants were invited to be re-interviewed within 3 months of the birth, in order to observe if views had changed.

The interview schedule and study materials were reviewed by a Patient and Public Involvement (PPI) representative (ex-smoker and current EC user). We also consulted a Smokers Panel of the UK Centre for Tobacco and Alcohol Studies for advice on the recruitment and research process. Interviews were conducted between October 2015 and October 2016. We used the Consolidated Criteria for Reporting Qualitative Research tool to promote a comprehensive report of the methods and findings [29].

### Recruitment and screening procedures

Participants were purposively sampled from the following groups: current EC users (used EC in previous 30 days), ex-users (used EC, but not in last 30 days); from each trimester of pregnancy, and from varying stages within 6 months postpartum. Various sites were used to place recruitment adverts: UK Stop Smoking Services (SSS), hospital antenatal clinics and health visitor clinics in locations across England and Scotland; social media (Facebook pages of the Nottingham Smoking in Pregnancy Research group (www.facebook.com/mumssmokingresearch/) and Tommy’s pregnancy charity (https://www.facebook.com/tommys/) and the PPI representative’s Twitter account); an advert on the pregnancy website Mumsnet (www.mumsnet.com); and through informal networks. Women attending SSS or clinics were informed of the study by a researcher or stop smoking advisor and contact details of those interested were sent to a researcher.

### Participant consent

Women selected for interview were sent a participant information sheet. Approximately 1 week later, a researcher telephoned to seek verbal informed consent to participate; pregnant women were asked for consent to contact them again postpartum. Women were ineligible if they could not understand the study procedure sufficiently or were under 16 years. Background questions included: age, ethnicity, gestation, smoking status, whether living with a partner, age left full-time education and employment. Ex and current EC users were asked about the frequency of EC use: (i) EC used daily (intensive users); (ii) ECs used regularly but not daily (intermittent users); (iii) ECs only used once or twice, or occasionally (triers).

### Interviews

Two experienced, female researchers conducted the interviews (KB: PhD, midwife, non-smoker and SO: PhD, health psychologist, non-smoker). The interviewers introduced themselves as researchers working at the University of Nottingham. Interviews were audio recorded. Data were transcribed verbatim by a transcriber external to the research team. Interviewees received a £15 shopping voucher. This payment was not offered on recruitment adverts.

Interview questions were adapted from previous topic guides related to ECs for smokers [30–33], with pregnancy-specific questions added. The main topics were: patterns of EC use (including motivations for using ECs), perceived social norms, views on ECs compared with cigarettes and NRT, views on support with using ECs and on legislation and advertising (see Additional files 1, 2, 3, 4, 5 and 6). Interview questions differed slightly according to participant’s experience of ECs. After interviewing 30 participants, and conducting initial coding, we reflected on whether data saturation had been reached. For those pregnant women who were interviewed a second time, during postpartum, we covered the same topics as in pregnancy and focussed on changes in views and experiences since the first interview. For the latter interviews, we interviewed all the women who were willing and we did not attempt to reach data saturation.

### Analysis

The framework method of thematic analysis was used to manage, summarize and analyse the data [34]. Researchers produced a set of framework matrices according to interviewees level of experience of ECs and whether they were pregnant or postpartum; thus, in total, there were six matrices (i.e., pregnant/current EC user, pregnant/ex-user, pregnant/never user, postpartum/current user, postpartum/ex-user, postpartum/never user). This enabled researchers to gain insight into the views and experiences of each participant, while also identifying any differences between participants according to their experience of ECs. Interviews from women who were interviewed twice (pregnancy and postpartum) were compared to explore if views had changed. The analysis was both inductive, from the accounts (experiences and views) of participants, and deductive, through being informed by existing literature as reflected in the study objectives and the topics chosen for the interviews.

First, KB and SO independently read and open coded all the transcripts line by line, referring to any notes made during the interviews. Also, in order to gain familiarity with the dataset, these two researchers listened to all the recordings. These researchers then met to discuss their respective codes and agree the codes and define code categories representing conceptually related codes that together formed a ‘working’ analytical framework. The transcripts were then independently coded a third time by either MU, FN, SC, LB or RW (all non-smokers), using the working framework and noting any codes that deviated from the framework. All the researchers then discussed possible refinements to the codes and categories. The revised analytical framework was then applied to each transcript to attach the final codes and to identify particularly meaningful extracts of text to be transferred to an Excel spreadsheet, consisting of one row per participant and a column per code. The matrix was then reviewed by KB and SO, with discussion with the entire team, to identify, label and refine the themes which best explained the data. To increase the integrity of the findings, attention was given to deviant cases. NVivo 11 software was used to assist with coding and analysis.

## Results

One hundred twenty-three women were eligible and interested in participating in the study; 30 (26%) were contactable and consented to an interview. Participants were recruited from: SSS (Glasgow (*n* = 4), Leicester (*n* = 7), London (*n* = 2)), Nottinghamshire antenatal clinic (*n* = 4) and health visitor clinics (*n* = 10), Tommy’s Facebook (*n* = 2), and informally (*n* = 1).

There were nine current-EC users, 11 ex-users and 10 never-users; 16 smoked and 14 were ex-smokers (see Table 1). Seven of the nine current EC users were dual users of cigarettes and ECs. Participants were aged 21 to 38 years, the majority were white British, lived with a partner, did not attend formal education beyond 18 years and were employed. Among the 15 pregnant women, three were in the first trimester (≤ 12 weeks gestation), seven in the second trimester (13 to 26 weeks), and five in the third (27–40 weeks). Fifteen women were postpartum: six were 0–3 months postpartum and nine were 4–6 months. Ten pregnant women gave consent to be re-interviewed postpartum and, of those, five were interviewed and five were uncontactable. Two of the participants changed their smoking/EC status by the second interview; one smoker/never EC user tried an EC since the interview and an ex-smoker/current EC user had returned to smoking. Interviews lasted for a mean (SD) 29 (8.7) minutes (range 18 to 53 min). We considered that data saturation and been reached following the initial 30 interviews.

**Table 1:** Characteristics of participants

ID	Smoking status	EC status	EC level of use	Age range	Weeks’ gestation/ months postpartum	Living with partner	Age left Education	Ethnicity	Employed	Interviewed second time Smoking status and EC
1	ex-smoker	ex-user	trier	30–39	37 weeks	yes	17	white British	yes	No change: smoker/ex EC user
2	ex-smoker	ex-user	trier	30–39	37 weeks	yes	16	white British	yes	
3	ex-smoker	never user	n/a	30–39	5 months postpartum	yes	21	Asian	yes	
4	ex-smoker	ex-user	trier	20–29	36 weeks	yes	18	white British	yes	
5	smoker	current user	intermittent	20–29	6 months postpartum	yes	18	white British	yes	
6	smoker	current user	intermittent	30–39	10 weeks	yes	26	white British	yes	
7	smoker	current user	intensive	20–29	4 months postpartum	yes	16	white British	yes	
8	smoker	current user	intensive	20–29	25 weeks	yes	19	white British	yes	
9	smoker	ex-user	intensive	20–29	27 weeks	no	16	white British	no	No change: smoker/ ex EC user
10	ex-smoker	current user	intensive	20–29	11 weeks	no	still in education	white British	yes	
11	smoker	never user	n/a	30–39	33 weeks	yes	18	white British	yes	
12	smoker	ex-user	intermittent	30–39	19 weeks	no	16	white British	no	
13	ex-smoker	never user	n/a	30–39	19 weeks	yes	16	white British	no	
14	ex-smoker	ex-user	trier	20–29	16 weeks	yes	17	white British	no	No change: ex-smoker/ ex EC user
15	smoker	never user	n/a	30–39	19 weeks	no	16	white British	yes	
16	ex-smoker	ex-user	trier	20–29	35 weeks	no	18	white British	yes	
17	smoker	never user	n/a	20–29	12 weeks	no	15	White/ black Caribbean	yes	Change: smoker/ ex EC user (trier)
18	smoker	ex-user	intensive	20–29	4 months postpartum	yes	16	white British	yes	
19	ex-smoker	current user	intermittent	20–29	21 weeks	yes	16	white British	yes	Change: Smoker/ current EC user
20	smoker	never user	n/a	20–29	4 months postpartum	no	15	white British	no	
21	ex-smoker	never user	n/a	20–29	4 months postpartum	yes	17	white British	yes	
22	smoker	current user	intermittent	20–29	1 month postpartum	no	23	black Caribbean	yes	
23	ex-smoker	ex-user	trier	20–29	< 1 month postpartum	yes	16	white British	yes	
24	smoker	current user	intensive	30–39	5 months postpartum	yes	22	white Asian	yes	
25	smoker	never user	n/a	20–29	3 months postpartum	yes	16	white British	yes	
26	smoker	current user	intensive	20–29	3 months postpartum	yes	15	white British	yes	
27	ex-smoker	ex-user	trier	20–29	4 months postpartum	yes	21	white British	yes	
28	ex-smoker	never user	n/a	20–29	3 months postpartum	no	21	White/ black Caribbean	yes	
29	ex-smoker	never user	n/a	20–29	< 1 month postpartum	yes	21	white British	yes	
30	smoker	ex-user	trier	20–29	4 months postpartum	yes	21	white British	yes	

The initial codes were very similar for each level of experience of using ECs as well as for pregnancy and postpartum women, except that aspects related to triggers for use and experiences of use were not relevant for never users. Therefore, it was decided that a common analytical framework would be developed, while noting any divergence for particular sub-groups of participants. The framework included six categories: social factors, general experience and usage, patterns of usage, comparison of ECs and NRT, perceived benefits of ECs and negative views on ECs; nested under these categories were a total of 32 codes (see Additional file 7). Review of the matrices, through discussion between the entire team, suggested that five main themes offered the best explanation of the data: motivations to use ECs, social stigma, using the EC, consumer aspects, and harm perceptions. The process of defining and labelling the themes was influenced by both the study objectives and by new concepts identified inductively from the data. Any differences in views according to level of experience of ECs or between pregnant and postpartum women are highlighted below. When pregnant women were interviewed for a second time at postpartum their views appeared unchanged except regarding the perceived stigma of ECs (see ‘Social Stigma’ theme).

### Motivations for use

Most women were motivated to quit smoking; some felt that ECs could help them quit and dual users of ECs and cigarettes said they felt that ECs helped them to reduce their smoking.

*‘I tried them but at the time* [prior to pregnancy] *I didn’t really have the motivation to stop properly but then, as I say, when I fell pregnant this time the first thing I did was buy an electronic cigarette’.*

***(06 antenatal smoker and current EC user)***

The majority indicated that they believed ECs were a less harmful alternative to smoking during pregnancy, reducing foetal exposure to toxins. Most women were aware that ECs usually contained nicotine but were unaware of other ingredients. Some said that ECs would reduce smoke odours on their clothes, in their home and on their children. A few felt ECs were safer than cigarettes for second-hand smoke exposure.

*‘it* [EC] *doesn’t pass on second-hand smoke, because even if the baby was close-by, which I wouldn’t have a baby close-by, it wouldn’t be dangerous’.*

**(19 antenatal ex-smoker and current EC user)**

Most interviewees felt ECs were a cheaper alternative to smoking. A few women were motivated to use an EC based on cost alone. In terms of what first triggered women to use ECs, some women were introduced to ECs, and encouraged to quit using ECs, by family and friends. A few tried ECs after recommendations from a health professional. Others reported experimenting out of curiosity, often on the ‘*Spur of the moment*’ **(09 antenatal ex-smoker and ex-EC user)** and these women generally reported they were not ready to quit, so didn’t pursue the EC.

*‘They* [family] *were a lot happier about me using that* [EC] *than obviously smoking. My Mum actually bought me the e-cigarette and she never ever bought me cigarettes in my life’.*

***(08 antenatal smoker and current EC user)***

A few women, having quit smoking, said they were more likely to use NRT than ECs if they relapsed, primarily due to the potential for ECs to steer them back to smoking, as a result of the similarity to smoking. Some ‘never’ and ex-users believed NRT would better support a quit attempt than ECs as regards ‘weaning off’ nicotine.

### Social stigma

Most women expressed feeling uncomfortable (both actual and perceived experience) about using ECs in public during pregnancy and, for some, also in postpartum, especially when with children. The women said they felt that they would be judged and perceived as a bad mother. Two women who were interviewed again after pregnancy indicated they felt more comfortable about the idea of using ECs after the pregnancy, believing that there was less public judgement and less risk to the baby at this time. Some women also expressed a strong belief that it was socially unacceptable for a pregnant woman to smoke or vape in public.

*‘If I was pregnant I would feel embarrassed because I think it looks disgusting. If I’m honest, pregnant women drinking or smoking any type of fags, cigar, electronic fags, I don’t think it looks very nice, so I wouldn’t do it pregnant in public. Afterwards I wouldn’t be bothered. I suppose it depends if I had the baby with me in a pushchair I wouldn’t be too keen on the idea.’*

***(04 antenatal ex-smoker and ex-EC user)***

Compared with cigarette smoking, some felt it was more socially acceptable to use an EC and felt empathetic towards pregnant women using ECs as they could relate to the struggles of cessation. A few mentioned that a benefit of NRT is that it can be administered discreetly in public.

### Using the EC (EC current users/ex-users only)

Regarding the context of where the EC was used, EC ‘triers’ reported vaping with friends and family, often in the home environment. Regular users generally reported vaping in similar environments as for smoking, often the home, workplace or car. A few said that, similarly to smoking, they avoided using ECs around their children.

Some said they were attracted by ECs as they replicated and substituted smoking, especially in terms of inhaling and blowing vapour and the hand-to-mouth action. One woman, who had struggled to quit using NRT, said she found that ECs helped her as they assisted with both the addiction and behavioural aspects of smoking. However, some ex-users indicated they felt ECs were too much of a reminder of smoking but, compared with a cigarette, didn’t provide the same satisfaction and often required use over longer periods in order to alleviate cravings.

*‘One thing I missed when I have quit smoking is inhaling the smoke, so when I used an e-cigarette obviously you’ve got that kind of experience of inhaling the vapour. It was too much, it was too similar to having a cigarette, so it made me miss it even more.’*

***(01 antenatal ex-smoker and ex-EC user)***

Some women reported negative first experiences of ECs; others felt side effects reduced as they adapted. One woman compared her initial experience to learning to smoke.

### Consumer aspects

Among EC users/ex-users there was a general preference for smaller and lighter ECs; one woman reported she liked the convenience of it fitting in her handbag. Most participants were aware of the variety of flavours and some were aware of various nicotine strengths. To economise, a few women purchased the cheapest EC; however, they also felt the price of ECs reflected their value, with more expensive devices being more user-friendly. Women tended to either choose an EC impulsively, based on cost, or based on the advice of friends/family. Many had not received instructions about their device from the retailer or on the EC packaging, leaving some feeling uninformed.

*‘When you are pregnant you want to know….how is the best way to use it* [EC] *to help you stop….you literally had to go and buy it like a loaf of bread’.*

***(07 postpartum smoker and current EC user)***

Rather than receiving information about ECs from retailers, women generally said they would prefer to receive information from a health professional or through National Health Service leaflets or websites.

### Harm perceptions

Never-users, in particular, and some ex-users, said they felt there was a lack of information available about the safety of ECs, which led to fears. In order to make an informed decision about whether to use ECs, they said they needed further information and advice, preferably from health professionals.

*‘Everyone just says ‘oh there’s only five chemicals in an e-cigarette, while there’s 4000 in a cigarette’ but my concern was those five chemicals that are in the e-cigarette’.*

***(18 postpartum smoker and ex-EC user)***

Women were asked whether they thought ECs should be made available on prescription and many felt it was important to first establish their safety. One smoker and never user said that she would require the government to provide assurance on their safety in pregnancy before she would consider using one.

*‘Yeah and I think obviously if there was some sort of government stamp on it or you know you don’t buy toys without having something, you don’t buy anything without, even the bad stuff you know, you buy a packet of cigarettes and the government have put what it can do to you on it, with all the pictures. Whereas there’s nothing is there? There’s no nothing, no good, no bad, no nothing.’*

**(15 antenatal smoker and never user)**

Some women indicated they felt more confident about NRT because the health service clearly advocated it, whereas they had received conflicting advice from health professionals about using ECs. Concerns about the safety of ECs were heightened by negative media reports, referring to malfunctioning devices, links to cancer or other health harms. A couple of women were concerned that ECs were becoming a fashion accessory, and one woman reported ECs had appealed to her young nephew.

Several women were worried that vaping might increase their consumption of nicotine compared with smoking:

*‘Obviously with a cigarette you can only smoke it for so long till it’s finished, but with an e-cigarette you can smoke for as long as you want to. So sometimes, I guess, I was taking in more than the usual nicotine intake that I would have done with a cigarette.’*

***(01 antenatal ex-smoker and ex-EC user)***

*‘So my friends that have used them they’re constantly in their mouth whereas a cigarette, you have a cigarette and then you don’t have another one for, say, three hours.’*

**(13 antenatal ex-smoker never user)**

## Discussion

This study explored in-depth views surrounding EC use in both pregnancy and postpartum, identified five main themes, and gained important new insights that are specific to this population. Some pregnant and postpartum women viewed ECs as less harmful than smoking and as aids for reducing and stopping smoking. However, due to perceived social stigma, some felt uncomfortable using ECs in public, especially during pregnancy. They also had concerns about safety and nicotine dependence, requesting more consistent information from health professionals. Some expressed a preference for NRT over ECs; they felt that NRT was both consistently sanctioned by health professionals and could be used more discretely than ECs.

Strengths of this study include the use of a broad-based topic guide and a series of individual interviews to capture the views of UK women with varying experience of ECs. Additionally, we recruited women from a number of UK regions and recruitment sites. Together, this approach increases the likelihood of capturing a diverse set of views. The use of framework analysis ensured data analysis was transparent and enabled all members of the research team to contribute.

There were also limitations. Telephone rather than face-to-face interviews were used; we were therefore unable to gather potentially important contextual information about the participant’s environment. However, considering both the wide geographical spread of the participants and that pregnancy is a busy time with many antenatal appointments, telephone interviews were considered the most practicable option. As regards reflexivity, there is the potential for unconscious, or even conscious, bias as this research was conducted in a UK research and policy climate in which ECs have largely been advocated as a less harmful alternative to smoking [11]. As with most qualitative research [35], the intention of the study was not to ensure the findings are fully representative of the wider population; although, the ethnic and education characteristics of women (i.e., mostly white ethnicity and not receiving formal education beyond 18 years) suggest that they are fairly typical of women who smoke in pregnancy [3]. Our intention was, purposively, to capture potential diversity around the phenomenon of views on and experiences of ECs during pregnancy and postpartum. Thus, as we purposively sampled for both level of experience with ECs and pregnancy versus postpartum, there was great heterogeneity in the characteristics of the sample. This can make it difficult to draw overall conclusions. Despite this, except for instances where experiences were irrelevant to the ‘never-users’ sub-group (e.g., regarding triggers for using ECs), the findings were very similar for the different sub-groups.

As with smokers in general [33], there were mixed findings concerning women’s experiences of using ECs; some favoured ECs because they mimicked smoking, while, conversely, others didn’t like ECs as they reminded them of smoking. Again, as found in the general population [36], most women were interested in using ECs either to stop smoking or to reduce their smoking with dual use of ECs and cigarettes. The majority (seven of nine) of the current EC users were dual users of ECs and cigarettes and these women said they felt that ECs helped them to reduce their smoking. Outside of pregnancy, 45% of British EC users report dual use [36]. The results of a recent nationally representative US survey suggest that rates of dual use may be higher in pregnancy than outside for pregnancy: 79% of 34 pregnant women using ECS were currently still smoking and the remaining 21% were ex-smokers [15]. Dual use of NRT and cigarettes is also common during pregnancy [37]. Dual use may increase attempts to stop smoking [12, 38], although it is unclear whether this increases cessation [39]. Among smokers in general, dual use is likely to maintain similar levels of carcinogens and toxicants as smoking [14], although it is not known if this is also the case in pregnancy.

Interestingly, many of the women said that they began using ECs spontaneously, ‘on the spur of the moment’. This may need to be considered in public health policy as ECs are increasingly widely used and available, and pregnant women may be frequently exposed to them and readily triggered to experiment with them.

This is the first study to report that, due to perceived social stigma, some women would avoid using ECs in public while pregnant or with an infant. Similar to US findings [26], others felt that using an EC was less stigmatising than smoking and would feel empathetic towards pregnant women using ECs, as they understood the difficulties of cessation. The presence of perceived social stigma contrasts with non-pregnant populations, where ECs are largely viewed as sociable and enjoyable [31, 39, 40], but is consistent with the strong social stigma felt by pregnant smokers [41–43] and with negative public perceptions of smoking in pregnancy [44]. Moreover, a US survey observed that most people view ECs as being as harmful, if not more harmful, as smoking during pregnancy [45]. Pregnant women hide their smoking [43] and, similarly, we observed that women often preferred vaping at home and in other places where they usually smoked. Moreover, there was no indication that women vaped with others or shared their ECs. These women may be responding to the societal focus on maternal responsibility and the notion of the ‘bad mother’, in relation to health threats to the fetus, which can serve to marginalise and stigmatise women [46]. Related to this, due to its discrete nature, some women favoured NRT over ECs; clinicians may need to be sensitive to this preference and EC manufacturers might want to consider producing more discrete devices that are not perceived to give a ‘bad’ impression. A further, related, new finding is that a few women felt it was more socially acceptable to use an EC after having a baby, compared with pregnancy, as they felt there was less stigma and less harm for the baby. Thus, ECs may be suitable for preventing return to smoking in the postpartum period, although it has also been observed that women who use ECs in pregnancy may be at risk of resuming smoking after the birth [26].

Consistent with US research among pregnant women [22, 25, 26, 47], many considered ECs to be safer than cigarettes during pregnancy and around infants. They also had concerns about harm and felt that health professionals needed to provide more consistent safety information. There were also worries that ECs continue or even increase nicotine addiction. Among non-pregnant smokers, laboratory analysis suggests it is unlikely vapers would consume more nicotine than when smoking, as they self-titrate to satisfy their cravings [48, 49]. The women’s need for more safety information may be partly alleviated by recent UK guidance to help healthcare professionals to advise pregnant women about ECs [19]. This guidance states the likely relative harms of ECs versus smoking and advises that NRT is the recommended option in pregnancy but that women should not be discouraged from using ECs if they help them to stay smoke free. Together, these findings highlight the urgent need for studies investigating EC toxicants and carcinogens for mother and fetus, similar to studies conducted with non-pregnant smokers [13].

## Conclusions

ECs were viewed positively by some pregnant and postpartum women and seen as less harmful than smoking and useful as aids for reducing and stopping smoking. However, due to perceived social stigma, some women feel uncomfortable using ECs in public, especially during pregnancy, and had concerns about safety and nicotine dependence. These findings highlight the need for both health professionals and designers of EC interventions to provide women with up-to-date and consistent information and advice, as well as considering the influence of social stigma. There remains a need for investigations of any harms of ECs for the mother and fetus and of the effectiveness of these devices for helping women to reduce and stop smoking during pregnancy and postpartum.

## Additional files

Additional file 1: Coding framework mind map. Brief description of the data: A mind-map figure, showing the six categories and 32 codes making up the coding framework. (DOCX 37 kb)

Additional file 2: Topic Guide pregnant users or used. Brief description of the data: Topic guide for women who are pregnant and are currently using ECs or have previously used them. (DOCX 30 kb)

Additional file 3: Topic Guide pregnant never used. Brief description of the data: Topic guide for women who are pregnant and have never used ECs. (DOCX 31 kb)

Additional file 4: Topic Guide postpartum users or used. Brief description of the data: Topic guide for women who are postpartum and are currently using ECs or have previously used them. (DOCX 28 kb)

Additional file 5: Topic Guide postpartum never used. Brief description of the data: Topic guide for women who are postpartum and have never used ECs. (DOC 56 kb)

Additional file 6: Topic Guide Follow up used. Brief description of the data: Topic guide for women for postpartum follow-up interview who in first interview (during pregnancy) said that they were currently using ECs or had previously used them. (DOCX 26 kb)

Additional file 7: Topic Guide Follow up never used. Brief description of the data: Topic guide for women for postpartum follow-up interview who in first interview (during pregnancy) said that they had never used ECs. (JPEG 66 kb)

## Abbreviations

EC: Electronic cigarette
NRT: Nicotine replacement therapy
