# Supplementary material for: Views on and experiences of electronic cigarettes: a qualitative study of women who are pregnant or have recently given birth
Source: BMC Pregnancy Childbirth. 2018 Jun 15;18:233. doi: 10.1186/s12884-018-1856-4 (PMC6003107; doi:10.1186/s12884-018-1856-4)
Supplement: Supplementary file 1 — Coding framework mind map. Brief description of the data: A mind-map figure, showing the six categories and 32 codes making up the coding framework. (DOCX 37 kb) [file 12884_2018_1856_MOESM1_ESM.docx]

**Topic Guide- Pregnant Users/Used E cigarettes**

**Introduction:** *Aim, to create appropriate atmosphere*

- Name of the interviewer and affiliation
- Purpose of the study, ensure PIS read and understood
- Consent to take part in the study
- Confidentiality, explain how the data will be used
- Interview will last approximately 30-40 minutes
- Audio recorded to ensure interviewer can fully engage in the interview

**Warm up questions:** *Aim, context about smoking and make women comfortable*

- Can you tell me how long you have/had been smoking?
- How do/did you feel about your smoking?
- Did becoming pregnant change your opinion about your smoking?
- Can you describe any attempts you have made to stop smoking during pregnancy?

**Knowledge of E Cigarettes:** *I would now like to spend some time discussing E cigarettes*

- Can you tell me how you first became aware of E cigarettes?
- What is your view on what is in an E cigarette?

Prompt *(if participant states ingredients that are in an e-cigarette) ask*

- Do you think these ingredients are harmful or not, particularly in pregnancy.
- What do you know about the different types of E cigarettes?

Prompt

- Can you describe what type/s of E cigarette you used
- What made you choose the E cigarette you used
- How do you feel about the different types of E cigarette devices?
- Can you tell me whether you smoked an e-cigarette before you became pregnant or was this something you used after becoming pregnant?

Prompt if yes,

- Did you notice any differences when using an E cigarette during pregnancy compared to before pregnancy? (i.e. experience/taste etc.)
- Before using an E cigarette, can you describe any concerns you had about using one?

**Patterns of E Cigarette use:**

- What led you to first start using E cigarettes?

Prompt

- Did someone recommend them? If so who?
- Did your pregnancy influence this decision?
- Were you influenced by someone else’s experience?
- Did health reasons influence your decision?
- Did cost of smoking influence your decision?
- Can you describe your first experience of using an E cigarette?

Prompt

- Can you describe how it first felt to use one?
- When did you first start using them (i.e. how along ago and before or during pregnancy)?
- How long did it take you to get used to it?
- Did anyone give you instructions on how to use it?
- When and where do/did you use it?
- When using an E Cigarette did you continue to smoke or use smoking cessation medication such as NRT at the same time?

Prompt

- Why? Did you plan to do this?
- What do you think are the positives and negatives to using an E cigarette?
- Currently you are pregnant, however after having your baby would you consider using/continue using an E Cigarette?

Prompt

- Can you tell me why you would/wouldn’t?
- Can you tell me why you think an E cigarette may/may not help you? (e.g., to quit, to cut down, safety)

*Former users of E cigarettes only: You reported that you have not used an E cigarette in the past 30 days.*

- Was there any particular reason why you stopped using E cigarettes?

Prompt

- Did you have any concerns?

*If participant requires more promoting:*

- Did someone tell you to stop*?*
- Was there anything you didn’t like about the device?
- Did you have any concerns you may become dependent on them?
- Did you experience any side effects?
- Did you have worries about the safety of E cigarettes for yourself or your baby?
- Was it because you heard bad press
- Did you manage to quit and felt you no longer needed them

**Social norms:**

- How do you feel using an E cigarette in public during pregnancy and how do you think you will feel in the months after having your baby?

- In your experience, how have others (friends and family) reacted to you or other pregnant women using an E cigarette during pregnancy or the months after having their baby?

Prompt

- Do you think their reaction would be different if you were not pregnant?

Do you think they find them more or less acceptable than smoking, or no difference?

If no experience of family/friends reactions

What kind of reaction do you expect from others towards using an E cigarette during pregnancy or the months after having their baby?

- Do you think people using E cigarettes in pregnancy might influence how many people smoke tobacco in pregnancy?

**Attitudes to E Cigarettes versus cigarettes:**

- What do you think of E cigarettes compared with cigarettes for smoking during pregnancy or during the months after the birth?

Prompt

- In comparison to cigarettes how safe do you think they are?
- In comparison to cigarettes how enjoyable is it?
- What do you see, if any, as the advantages of E cigarettes over cigarettes during pregnancy?

Prompt

- Do you think electronic cigarettes are more or less safe for yourself and your baby compared with cigarettes?
- Do you think other people are more positive or more negative about using E Cigarettes during pregnancy compared to cigarettes?
- Do E cigarettes taste better or worse than a cigarette?
- Are E cigarettes more or less satisfying than cigarettes?
- How did/do you find the sensation when inhaling an E cigarette?
- How did/do you find the taste?
- Are there any cost differences?
- What do you see as the disadvantages of E cigarettes compared with cigarettes?

Prompt

- Safety/effectiveness/substances
- Not supported by NHS/SSSs
- Bad media press
- Not knowing what advice to believe/the best place to buy them / if retail shop /online store can be trusted
- Did/does your E cigarette come with different flavours?

Prompt

- What flavours do/did you prefer and why?
- What flavours did not appeal and why?
- Did you like having a choice of flavours and why?
- What do you think about using E Cigarettes while you are still smoking, to help cut down during pregnancy or after having your baby?

**Attitudes to E Cigarettes compared with NRT**

- Can you tell me your views on nicotine replacement therapy (NRT)
- What do you think of E cigarettes compared with nicotine replacement therapy?

Prompt

- To help to stop or reduce your smoking, would you prefer to use nicotine patches, oral NRT products (e.g. inhalator or gum), or E cigarettes?
- In comparison to NRT, how helpful do you think E Cigarettes are/or might be for helping you to stop smoking, avoid going back to smoking after the birth, or to reduce the amount you smoke?
- What do you see as the advantages of E cigarettes over NRT? (e.g., less perceived stigma, do not have skin aggravation of patches, prefer taste of E Cigarettes, E Cigarettes are less medical)
- What do you see as the disadvantages of E cigarettes compared with NRT? (e.g., less known about risks of E Cigarettes)
- Do you think one may be better at helping people stop smoking?
- Do you think one may be safer?
- What do you think about the idea of using E cigarettes together with a nicotine patch?

**Support for using E Cigarettes**

- Where do/did you buy your E cigarette from? How do you feel about that?
- When you first bought your E cigarette can you remember what information the seller gave you about it?

Prompt

- How useful was this advice?

Have you been offered or sought advice or support on using them from anywhere else? (e.g. health professional, family, friends, internet, online forums, SSSs)

How useful was this?

- Would you like the seller to give you advice about how to use E Cigarettes and about any risks of using them during or soon after pregnancy?
- How would you like the advice about E cigarettes to be given to you? (e.g., leaflet only, brief advice alone, leaflet plus brief advice, website information)

**Legislation and advertising**

- What do you think about E Cigarettes being offered more as a medical product, more like nicotine patches?
- Do you think they should be offered on prescription?
- What do you think about the idea that E Cigarettes are not recommended for use during pregnancy?
- Have you come across any advertisements for E cigarettes? If so what do you think of them?

**Summary**

- Briefly clarify the main ideas that have been discussed in the interview and check with the participant whether the summary is accurate
- Thank participant for taking part
