# Supplementary material for: Views on and experiences of electronic cigarettes: a qualitative study of women who are pregnant or have recently given birth
Source: BMC Pregnancy Childbirth. 2018 Jun 15;18:233. doi: 10.1186/s12884-018-1856-4 (PMC6003107; doi:10.1186/s12884-018-1856-4)
Supplement: Supplementary file 3 — Topic Guide pregnant never used. Brief description of the data: Topic guide for women who are pregnant and have never used ECs. (DOCX 31 kb) [file 12884_2018_1856_MOESM3_ESM.docx]

**Topic Guide- Postpartum Users/Used E cigarettes**

**Introduction:** *Aim, to create appropriate atmosphere*

- Name of the interviewer and affiliation
- Purpose of the study, ensure PIS read and understood
- Consent to take part in the study
- Confidentiality, explain how the data will be used
- Interview will last approximately 30-40 minutes
- Audio recorded to ensure interviewer can fully engage in the interview

**Warm up questions:** *Aim, context about smoking and make women comfortable*

- Can you tell me how long you have/had been smoking?
- How do you feel about your smoking?
- Did becoming pregnant or having your baby change your opinion about your smoking?
- Can you describe any attempts you have made to stop smoking during pregnancy or following the birth of your baby?

**Knowledge of E Cigarettes:** *I would now like to spend some time discussing E cigarettes*

- Can you tell me how you first become aware of E cigarettes?
- What is your view on what is in an E cigarette?

Prompt *if participant states ingredients that are in an e-cigarette ask*

- Do you think these ingredients are harmful or not, particularly in pregnancy.
- What do you know about the different types of E cigarettes?

Prompt

- Can you describe what type of E cigarette you used
- What made you choose the E cigarette you used
- How do you feel about the different types of E cigarette devices?
- Can you tell me whether you used an E cigarette before, during or after pregnancy?

Prompt if yes,

- Did the experience of using an E cigarette or smoking change for you?
- Did the taste of the E cigarette or smoking change after becoming pregnant? If yes, please tell me in what way?
- Before using an E cigarette, can you describe any concerns you had about using one?

**Patterns of E Cigarette use:**

- What led you to first start using E cigarettes?

Prompt

- Did someone recommend them? If so who?
- Did your pregnancy or baby influence this decision?
- Were you influenced by someone else’s experience
- Did health reasons influence your decision?
- Did cost of smoking influence your decision?
- What were your main reasons for wanting to use E Cigarettes?

(e.g., baby/myself than cigarettes, cheaper than cigarettes)

- Can you describe your first experience of using an E cigarette?

Prompt

- Can you describe how it first felt to use one?
- How long did it take you to get used to it?
- Did anyone give you instructions on how to use it?
- When and where do/did you use it?
- When using an E Cigarette did you continue to smoke or use smoking cessation medication such as NRT at the same time?

Prompt

- Why? Did you plan to do this?
- What do you think are the positives and negatives to using an E cigarette?

*Former users of E cigarettes only: You reported that you have not used an E cigarette in the past 30 days.*

- Was there any particular reason why you stopped using E cigarettes?

Prompt

- Did someone tell you to stop*?*
- Was there anything you didn’t like about the device?
- Did you have any concerns you may become dependent on them?
- Did you experience any side effects?
- Did you have worries about the safety of E cigarettes?

**Social norms:**

- How comfortable do you feel using an e-cigarette in public during pregnancy or the months after having your baby?

- In your experience, how have others (friends and family) reacted to you or other pregnant women using an E cigarette during pregnancy or the months after having their baby?

Prompt

- Do you think their reaction would be different if the woman was not pregnant?

Do you think they find them more or less acceptable than smoking, or no difference?

- In recent years smoking cigarettes has become less popular and some people think that E cigarettes may make smoking in pregnancy popular again. What do you think about this idea?

**Attitudes to E Cigarettes versus cigarettes**

- What do you think of E cigarettes compared with cigarettes for smoking during pregnancy or during the months after the birth?

Prompt

- In comparison to cigarettes how safe do you think they are?
- In comparison to cigarettes how enjoyable is it?
- What do you see, if any, as the advantages of E cigarettes over cigarettes?

Prompt

- Do you think electronic cigarettes are more or less safe for yourself and your baby compared with cigarettes?
- Do you think other people are more positive or more negative about using E Cigarettes during pregnancy or after having a baby compared to cigarettes?
- Do E cigarettes taste better or worse than a cigarette?
- Are E cigarettes more or less satisfying than cigarettes?
- How did/do you find the sensation when inhaling an E cigarette?
- How did/do you find the taste?
- Are there any cost differences?
- What do you see as the disadvantages of E cigarettes compared with cigarettes?

Prompt

- Safety/effectiveness/substances
- Not supported by NHS/SSSs
- Bad media press
- Not knowing what advice to believe/the best place to buy them / if retail shop /online store can be trusted
- Did/does your E cigarette come with different flavours?

Prompt

- What flavours do/did you prefer and why?
- What flavours did not appeal and why?
- Did you like having a choice of flavours and why?
- What do you think about using E cigarettes after having your baby compared to smoking?
- What do you think about using E Cigarettes while you are still smoking, to help cut down during pregnancy or after having your baby?

**Attitudes to ECs compared with NRT**

- Can you tell me your view on nicotine replacement therapy (NRT)
- What do you think of E cigarettes compared with nicotine replacement therapy?

Prompt

- To help to stop or reduce your smoking, would you prefer to use nicotine patches, oral NRT products (e.g. inhalator or gum), or E cigarettes?
- In comparison to NRT, how helpful do you think E Cigarettes are/or might be for helping you to stop smoking, avoid going back to smoking, or to reduce the amount you smoke?
- What do you see as the advantages of E cigarettes over NRT? (e.g., less perceived stigma, do not have skin aggravation of patches, prefer taste of E Cigarettes, E Cigarettes are less medical)
- What do you see as the disadvantages of E cigarettes compared with NRT? (e.g., less known about risks of E Cigarettes)
- Do you think one may be better at helping people stop smoking?
- Do you think one may be safer?
- What do you think about the idea of using E cigarettes together with a nicotine patch

**Support for using E Cigarettes**

- Where do/did you buy your E cigarette from? How do you feel about that?
- When you first bought your E cigarette can you remember what information the seller gave you about it?

Prompt

- How useful was this advice?

Have you been offered or sought advice or support on using them from anywhere else? (e.g. health professional, family, friends, internet, online forums, SSSs)

How useful was this?

- Would you like the seller to give you advice about how to use E Cigarettes and about any risks of using them during or soon after pregnancy?
- How would you like the advice about E cigarettes to be given to you? (e.g., leaflet only, brief advice alone, leaflet plus brief advice, website information)

**Legislation and advertising**

- What do you think about E Cigarettes being offered more as a medical product, more like nicotine patches?
- Do you think they should be offered on prescription?
- What do you think about the idea that E Cigarettes are not recommended for use during pregnancy?
- Have you come across any advertisements for E cigarettes? If so what do you think of them?

**Summary**

- Briefly clarify the main ideas that have been discussed in the interview and check with the participant whether the summary is accurate
- Thank participant for taking part
