# Supplementary material for: Views on and experiences of electronic cigarettes: a qualitative study of women who are pregnant or have recently given birth
Source: BMC Pregnancy Childbirth. 2018 Jun 15;18:233. doi: 10.1186/s12884-018-1856-4 (PMC6003107; doi:10.1186/s12884-018-1856-4)
Supplement: Supplementary file 4 — Topic Guide postpartum users or used. Brief description of the data: Topic guide for women who are postpartum and are currently using ECs or have previously used them. (DOCX 28 kb) [file 12884_2018_1856_MOESM4_ESM.docx]

**Topic Guide- Postpartum Never used E cigarettes**

**Introduction:** *Aim, to create appropriate atmosphere*

- Name of the interviewer and affiliation
- Purpose of the study, ensure PIS read and understood
- Consent to take part in the study
- Confidentiality, explain how the data will be used
- Interview will last approximately 30-40 minutes
- Audio recorded to ensure interviewer can fully engage in the interview

**Warm up questions:** *Aim, context about smoking and make women comfortable*

- Can you tell me how long you have/had been smoking?
- How do you feel about your smoking?
- Did becoming pregnant or having your baby change your opinion about your smoking?
- Can you describe any attempts you have made to stop smoking during pregnancy or following the birth of your baby?

**Knowledge of E Cigarettes:** *I would now like to spend some time discussing E cigarettes*

- Can you tell me what you know about E cigarettes

Prompt

- What do you think they do?
- What do you know about the different types of E cigarettes?
- Do you know where you can buy E cigarettes from?
- How did you first become aware of E cigarettes?
- What are your views on what is in an E cigarette?
- What do you think are the advantages / disadvantages of using an E-cigarette? (e.g., healthy, safety, cost, flexibility, spending more time with children, embarrassing to use in public, inconvenient to use, not satisfying enough)

**Attitudes towards using E cigarettes:**

- You report that you have not used an E cigarette before. Is there any particular reason why?

Prompt

- Did someone tell you to stop*?*
- Was there anything you didn’t like about the device?
- Concerns about side effects?
- Health concerns?
- Did you have any other concerns? (i.e. may become dependent on them)
- Do you think you would consider using an E Cigarette in the future?
- If no can you tell me why (probe for design, functionality, cost, safety), what would convince you to use them (probe for safety)
- If yes can you tell me why (probe for when they would use it, functionality, design, safety)

**Social norms:**

- How comfortable would you feel using an e-cigarette in public during pregnancy or the months after having your baby?

- How do you think your friends and family would react to you or other pregnant women using an E cigarette during pregnancy or the months after having their baby?

Prompt

- Do you think their reaction would be different depending on whether you were pregnant?

Do you think they find E cigarettes more or less acceptable than smoking, or no difference?

- Do you think people using E cigarettes in pregnancy might influence how many people smoke tobacco in pregnancy?

**Attitudes to E Cigarettes versus cigarettes**

- What do you think of E cigarettes compared with cigarettes for smoking during pregnancy or during the months after the birth?

Prompt

- In comparison to cigarettes how safe do you think they are?
- In comparison to cigarettes how enjoyable is the experience?
- What do you see, if any, as the advantages of E cigarettes over cigarettes?

Prompt

- Do you think electronic cigarettes are more or less safe for yourself and your baby compared with cigarettes?
- Do you think other people are more positive or more negative about using E Cigarettes during pregnancy and in the months after having a baby compared to cigarettes?
- Do you think E cigarettes are more or less satisfying than cigarettes?
- What do you see as the disadvantages of E cigarettes compared with cigarettes?
- Safety/effectiveness/substances
- Not supported by NHS/SSS
- Bad media press
- Not knowing what advice to believe/the best place to buy them / if retail shop /online store can be trusted
- What do you think about using E cigarettes now you have had your baby compared to smoking?
- What do you think about using E Cigarettes while you are still smoking, to help cut down during pregnancy or now you have had your baby?

**Attitudes to E Cigarettes compared with NRT**

- Can you tell me what you know about nicotine replacement therapy (NRT)

- What do you think of E cigarettes compared with nicotine replacement therapy?

Prompt

- To help to stop or reduce your smoking, would you prefer to use nicotine patches, oral NRT products (e.g. inhalator or gum), or E cigarettes?
- In comparison to NRT, how helpful do you think E Cigarettes are/or might be for helping you to stop smoking, avoid going back to smoking after the birth, or to reduce the amount you smoke?
- What do you see as the advantages of E cigarettes over NRT? (e.g., less perceived stigma, do not have skin aggravation of patches, prefer taste of E Cigarettes, E Cigarettes are less medical)
- What do you see as the disadvantages of E cigarettes compared with NRT? (e.g., less known about risks of E Cigarettes)
- Do you think one may be better at helping people stop smoking?
- Do you think one may be safer?
- What do you think about using E cigarettes together with a nicotine patch?

**Support for using E Cigarettes**

- If you were to receive information about E cigarettes what information would you like to be told about?

Prompt

- Would you like the seller to give you advice about how to use an E Cigarettes and about potential risks for using them during or soon after pregnancy?
- How would you like advice about E cigarettes to be given to you? (e.g., leaflet only, brief advice alone, leaflet plus brief advice, website information)

**Legislation and advertising**

- What do you think about E Cigarettes being offered more as a medical product, more like nicotine patches?
- Do you think they should be offered on prescription?
- What do you think about the idea that E Cigarettes are not recommended for use during pregnancy?
- Have you come across any advertisements for E cigarettes? If so what do you think of them?

**Summary**

- Briefly clarify the main ideas that have risen from the interview and check with the participant whether the summary is accurate
- Thank participant for taking part
