# Supplementary material for: Views on and experiences of electronic cigarettes: a qualitative study of women who are pregnant or have recently given birth
Source: BMC Pregnancy Childbirth. 2018 Jun 15;18:233. doi: 10.1186/s12884-018-1856-4 (PMC6003107; doi:10.1186/s12884-018-1856-4)
Supplement: Supplementary file 5 — Topic Guide postpartum never used. Brief description of the data: Topic guide for women who are postpartum and have never used ECs. (DOC 56 kb) [file 12884_2018_1856_MOESM5_ESM.doc]

**Topic Guide- Follow up former EC users/used during pregnancy**

Key points from 1st interview

- Date of interview /gestation at time of interview:
- Smoking status/behaviour:
- Pattern of EC use etc.

**Introduction:** *Aim, to create appropriate atmosphere*

- Name of the interviewer and affiliation
- Purpose of the second ‘follow up’ interview, ensure PIS read and understood
- Consent to take part in the study
- Confidentiality, explain how the data will be used
- Interview will last approximately 30-40 minutes
- Audio recorded to ensure interviewer can fully engage in the interview

Warm up/context

How many weeks old is your baby now?

How have you been since baby arrived?

**Changes since last interview:** *Aim, context about smoking and make women comfortable*

- How have things been with your smoking/not smoking since we last spoke?
- Have you used an E cigarettes since we last spoke (both during the rest your pregnancy and since the birth)?

If yes

Prompt

- What are your reasons for using them now?
- In what circumstances are you using them (e.g. to avoid exposing baby to smoke in home)
- Is your experience of using an e-cigarette different since having your baby?
- Did having your baby make you feel any different about your smoking, use of E cigarettes or NRT compared with when you were pregnant?

Prompt

- If yes, what were you reasons for using an E cigarette after having your baby?
- If no, what were your reasons for not?
- Since having your baby, has your opinion of E cigarettes changed?

Prompt

If yes- Can you tell me how? What has changed your opinion?

If no- Can you describe your current opinion of E cigarettes?

- Can you tell me about any attempts you have made to stop smoking since we last spoke?

**Knowledge of E Cigarettes:** *I would now like to spend some time discussing E cigarettes*

- Have you heard any more about ECs since we last spoke?

**Patterns of use of E Cigarettes:**

*Current E cigarette users only*

- While you were pregnant, did you intend to use an E-cigarette after having your baby?

Prompt

- Why was this? (i.e. health reasons, influence of other people)

*Former E cigarette users only*

- Can you tell me why since we last spoke you have stopped using E Cigarettes, what was the reason for this:

Prompt

- Did someone suggest you should stop*?*
- Was there anything you didn’t like about the device?
- Did you have any concerns you may become dependent on them?
- Did you experience any side effects?
- Did you have worries about the safety of E cigarettes?

*Everyone*

• What do you think are the advantages / disadvantages of using an E-cigarette after having a baby? (e.g., health, safety, cost, flexibility, spending more time with children, embarrassing to use in public, inconvenient to use, not satisfying enough, to protect baby from second-hand smoke in home or car)

**Social norms:**

- How comfortable do/would you feel about using an E-cigarette in public now you have had your baby?

Prompt

- Do you feel differently about using an e-cigarette in public now you are not pregnant?

- How do/would your friends and family react to you using an E cigarette now you have had your baby?

Prompt

- Do you think their reaction is different now you are not pregnant?

**Attitudes to E Cigarettes versus NRT/cigarettes**

- Having had your baby, what do you think of E cigarettes compared with cigarettes or NRT?

Prompt

- Do you think electronic cigarettes are more or less safe for yourself and your baby compared with cigarettes?
- In comparison to cigarettes/NRT how enjoyable is the experience?
- What do you think about using E Cigarettes around your baby?
- Do you think that E Cigarettes are helping you to not go back to smoking or to cut down?
- If you are using E Cigarettes and smoking – when do you use ECs and when do you smoke?
- Has the SSS given you any support with trying to avoid smoking at the end of pregnancy or after the birth?
- Now you have had your baby, what do you think about using E Cigarettes while you are still smoking, to help cut down after having a baby?

**Summary**

- Briefly clarify the main ideas that have risen from the interview and check with the participant whether the summary is accurate
