# Supplementary figures and images for: Views on and experiences of electronic cigarettes: a qualitative study of women who are pregnant or have recently given birth
Source: BMC Pregnancy Childbirth. 2018 Jun 15;18:233. doi: 10.1186/s12884-018-1856-4 (PMC6003107; doi:10.1186/s12884-018-1856-4)

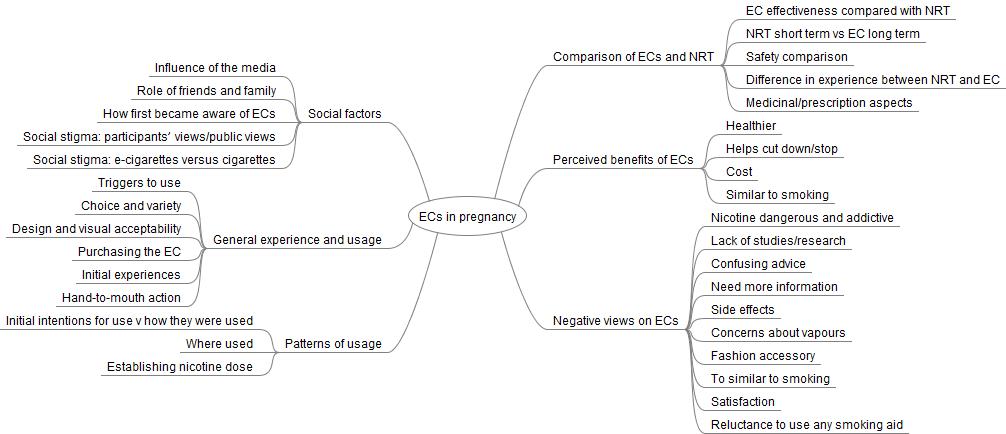

Supplement: Supplementary file 7 — Topic Guide Follow up never used. Brief description of the data: Topic guide for women for postpartum follow-up interview who in first interview (during pregnancy) said that they had never used ECs. (JPEG 66 kb) [file 12884_2018_1856_MOESM7_ESM.jpeg]
